# Design Analysis of Migration Nozzles Using CFD

**DOI:** 10.3390/polym17202766

**Published:** 2025-10-15

**Authors:** Makhsuda Juraeva, Dong-Jin Kang

**Affiliations:** School of Mechanical Engineering, Yeungnam University, 280 Daehak-ro, Gyoungsan 38541, Republic of Korea; mjuraeva@ynu.ac.kr

**Keywords:** migration nozzle, yarn channel, air orifice, vorticity, CFD

## Abstract

This paper presents a design analysis approach for migration nozzles used in the spinning process of synthetic fibers. A migration nozzle system consists of a yarn channel, air orifices, and a yarn loading slit. The entire system was analyzed in detail using computational fluid dynamics (CFD). The design parameters considered include the cross-sectional shape of the yarn channel, as well as the diameter and number of air orifices. Two different cross-sectional shapes, square and circle, were examined. The diameter of the air orifice varied from 0.6 mm to 2.0 mm, and both single and double orifice configurations were studied. A square cross-section resulted in the formation of a secondary vortex above the main vortex, making the circular cross-section preferable. The diameter of the air orifice significantly affects the vortex flow within the yarn channel. Vortex flow characteristics were quantified in two ways: the vorticity averaged across the cross-section in the direction of the yarn channel and the vorticity at the centerline. The highest vorticity at the centerline was observed at a diameter of 1.3 mm for single air orifice and 0.9 mm for double air orifices. These CFD results were validated through comparison with corresponding experimental data. A statistical analysis confirms that the centerline vorticity, particularly in the area of the air orifice, is a key and reliable parameter for evaluating the design of migration nozzles.

## 1. Introduction

Synthetic fibers are commonly produced using air-jet spinning technology, which utilizes various types of nozzles. These nozzles are designed to create a turbulent and non-uniform air flow environment that entangles and twists the yarns. Consequently, the formation of stable yarns is heavily dependent on the air flow pattern within the nozzle. This air-jet textured yarn spinning technology originated with a patent by the Dupont company in 1951 [1]. Initially, research and development efforts focused on increasing yarn production speeds, which were approximately 50 m/min in 1950s. By 1997, the Heberlain company had increased this production speed to 800 m/min [1].

Recent research and development efforts are focused on producing high-quality yarns through advanced jet design. This design initiatives require a comprehensive understanding of the airflow within an air-jet nozzle, taking into account of various parameters such as the number of air jet orifices, nozzle pressure, the diameter of air-jet orifice and the cross-section shape of the nozzle. For example, Murata Machinery Limited introduced the Murata Jet Spinning (MJS) 2.3 in 1997 [2]. This technology generated high speed air flows and demonstrated several advantages in the yarns produced, such as well-separated, straightened, and parallel fibers with minimal damage. When this airflow pattern is maintained at high production speeds, yarn productivity can increase to approximately 20 times that of ring spinning and 2.5 times that of rotor spinning technology. According to Alam et al. [2], air-jet vortex yarns showed 90% less hairiness compared to ring-spun yarns. Consequently, the air-jet vortex yarn technology holds significant innovative efficiency potential in the textile industry.

Significant research efforts and numerous papers have focused on effectively implementing air-jet spinning in nozzle design for the yarn production industry. For example, Ortlek and Ulku [3] investigated the effects of various parameters, such as delivery speed and nozzle pressure on the properties of vortex yarns produced using a Murata vortex spinning system. They reported that increasing the delivery speed leads to higher hairiness and fewer knots, while reducing the tensile properties of vortex yarns. Basal et al. [4] examined the effects of several process parameters, including nozzle angle, nozzle pressure, spindle diameter, yarn delivery speed, and the distance between the front roller and the spindle, on the structure and properties of vortex spun yarns. Zhong et al. [5] conducted experimental studies on the structure of air-jet vortex spun yarn and demonstrated that the yarn consists of two layers: the inner layer contains approximately 30% of fibers that are untwisted or loosely twisted, while the outer layer comprises fibers twisted in a helical form.

Textile nozzles used in air-jet spinning technology can be categorized into three distinct groups based on their applications: air-jet interlacing, air-jet twisting, and air-jet migration. Although the air-jet flow characteristics of these three types involve generating an air vortex flow inside a nozzle, each air-jet spinning nozzle must be designed to meet the specific requirements of the yarn production industry.

The air-jet interlacing nozzle represents the early version of air-jet spinning technology, introduced by DuPont in 1961. Since its invention, extensive research has focused on the performance of air-jet texturing nozzles. For instance, Chau et al. [6] employed a numerical approach to predict the yarn interlacing frequency of a triangular interlacing nozzle. Utilizing an inviscid, compressible flow model, they developed a mathematical model to predict the yarn interlacing frequency, which is primarily influenced by factors such as vorticity strength along the yarn feeding direction, air density, yarn density, and yarn feed speed. Juraeva et al. [7] proposed a design approach to optimize an air interlacing nozzle by using a compressible turbulent flow model, taking into account the shape of the yarn channel. They demonstrated that maximizing vorticity strength along the yarn feeding direction is directly associated with increased knot formation in the yarn.

The air-jet twisting nozzle is extensively used in the Spandex yarn industry as part of the winding and unwinding processes. It allows two or more spun yarns to be twisted together to achieve the desired yarn thickness [8]. Additionally, the nozzle helps maintain uniform tension of the filament during manufacturing [7]. Several researchers have investigated the air-jet vortex mechanism to enhance the performance of air-twisting nozzles. For example, Zheng et al. [9] developed an analytical model to express the strength of the vortex twist as a function of factors such as air pressure, flow rate, jet orifice angle, and jet orifice diameter. Furthermore, Zheng et al. [10] utilized a two-phase flow model to simulate fiber motion within the nozzle of an air-jet spinning machine, examining how parameters like air pressure, jet orifice angle, and fiber flexural rigidity affect wrapper formation, and consequently, yarn structure and quality. Juraeva et al. [11] simulated the vortex flow inside air-jet twisting nozzles under various operating conditions, evaluating the efficiency of the nozzle in terms of vorticity strength and uniformity. Since vortex flow is highly dependent on the geometry of air-jet texturing nozzles, a comprehensive study is needed to identify the most significant design parameter that remains consistent across various geometric variations.

Textile migration nozzles are utilized during the post-spinning stage of synthetic fiber production. Fiber migration involves the cyclic movement of fibers within a yarn channel during the spinning process [12,13]. To facilitate this cyclic movement, a small amount of oil mist is typically added. This migration is primarily influenced by tension differences between fibers at various radial positions, as well as the twisting air vortex flow, while minimizing friction and damage. Consequently, the migration process impacts yarn properties such as strength, number of knots, elongation, and appearance. When designing migration nozzles, it seems crucial to consider the air-jet vortex flow inside the nozzle. However, textile migration nozzles have not been extensively studied, particularly in terms of the airflow characteristics within the nozzle. Despite the presence of numerous geometric and operational parameters, such as air orifice, yarn channel, manufacturing tolerance and air pressure, these ultimately influence the flow characteristics within the yarn channel. Currently, it is unclear which specific flow characteristic parameter plays a crucial role in the design of migration nozzles.

The objective of this study is to simulate the airflow characteristics within a migration nozzle and propose a practical approach for the design analysis of migration nozzles using Computational Fluid Dynamics (CFD). A typical migration nozzle consists of a yarn channel, an air orifice, and a yarn loading slit [14]. Compressed air is supplied to the migration nozzle through the air orifice. Consequently, this study examines the effects of various factors such as the shape of the yarn channel cross-section, air orifice diameter, the number of air orifices. The computational domain of the migration nozzle was analyzed using ANSYS 2021R2 [15]. The performance of the migration nozzle is evaluated based on airflow velocity and axial vorticity. A parametric study was conducted to explore the effects of yarn channel shape and the number and diameter of air orifices. An optimized nozzle was fabricated to validate the numerical simulation results in terms of the number of yarn knots and the strength of spun yarn.

## 2. Migration Nozzle System

**Figure 1:**
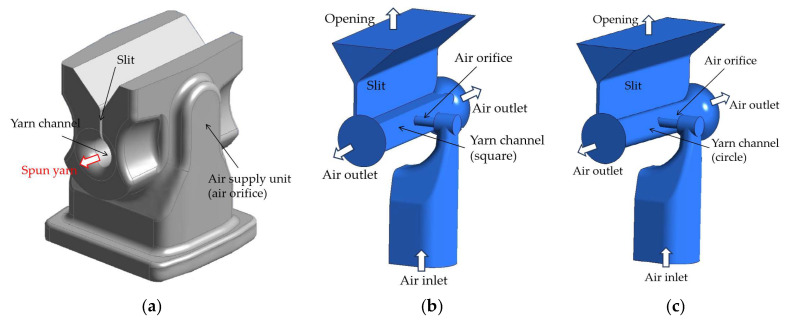
A computational sketch of the migration nozzle: (**a**) Housing; (**b**) The computational domain with a square yarn channel; (**c**) The computational domain with a circular yarn channel.

**Figure 2:**
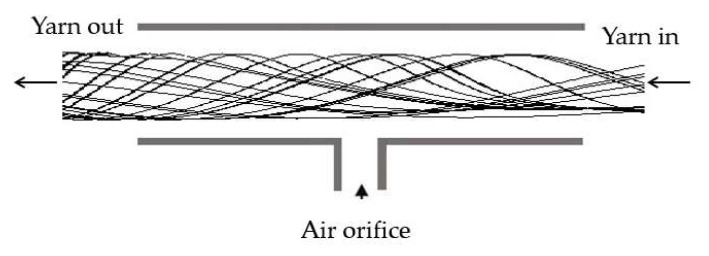
The basic migration nozzle (horizontal cut view).

Figure 1 illustrates a computational sketch of a housing with the current migration nozzle and provides a cross-sectional view. The computational model for the migration nozzle system comprises air orifices, a yarn channel, and a yarn loading slit. In this study, two different yarn channel shapes—square and circular—were simulated to analyze their influence on the air vortex characteristics within the channel. The square channel features a total length of 12 mm, with both width and height measuring 2 mm. The circular yarn channel is designed with a diameter of 2.0 mm, as detailed in Table 1. The air orifice is circular with a diameter of 0.6 mm, and the duct is oriented perpendicularly to the yarn channel. The yarn loading slit consists of two sections: a straight slit with a rectangular cross-section and a width of 0.2 mm, and a diverging slit connected to the straight slit, whose width increases linearly to 4.0 mm over a distance of 2.5 mm [16]. Figure 2 presents a horizontal cut view of the nozzle with yarns for the scenario of a single air orifice. These geometric configurations are designed to replicate the physical setup used in synthetic yarn spinning.

In the migration nozzle system, compressed air is introduced through the air orifice, impacting the opposite wall of the yarn channel and generating a swirling motion around the air orifice. This air then exits through both ends of the yarn channel. As the compressed air travels through the channel, it transports a small amount of oil mists, which serve to evenly coat the yarns. However, the size and quantity of these oil mists are negligibly small, so oil mist was not included in this computational analysis. The loading slot serves two conflicting purposes: providing fibers through it and minimizing pressurized air leakage. Consequently, the geometry of the loading slot is included in this simulation study. However, its effects are not examined in detail in this paper.

## 3. Computational Approach

In this study, all simulations were performed using the commercial software ANSYS 2021R2 [15]. ANSYS Workbench [15] was utilized to generate the computational domain and mesh for the simulations. CFX-Post [15] facilitated the analysis of the simulation results.

The governing equations for this study are the Reynolds-averaged steady Navier–Stokes (RANS) equations, with the Shear Stress Transport (SST) model applied as the turbulence model. The SST turbulence model is recognized for its high accuracy in scenarios with adverse pressure gradients and flow separation, as it incorporates transport effects into the eddy-viscosity formulation [16,17]. Specifically, it combines the strengths of the k-ω (Wilcox) model in the near-wall region and the k-ε (Launder and Spalding) model in the free stream, it is widely used as a two-equation eddy-viscosity model in CFD [18]. Since vortex flows are expected to form inside the yarn channel, the SST turbulence model would be generally a better choice in predicting flow separation under adverse pressure gradients and offers improved accuracy compared to individual k-ω or k-ε models. For example, Adanta et al. [19] compared the k-ε model and the SST model in the flow analysis of a breastshot waterwheel. The SST model was found to better predict the turbulent features such as the eddy viscosity and the turbulence intensity. Nevertheless, there are some conflicting arguments regarding the accuracy of these two-equation turbulence models, as it depends on the specific flow characteristics [20].

The boundary conditions for the computational domains depicted in Figure 1b,c are summarized in Table 2. The nozzle body is defined as a wall boundary, the outlet of the yarn loading slit is designated as an opening, and the end section of the air orifice serves as the inlet. All wall boundaries are treated as adiabatic surfaces with a no-slip condition, meaning there is no heat transfer across these surfaces and the fluid velocity at the wall is zero. At the inlet, the total pressure of 3 bar and total temperature of 20 °C were defined. This is standard practice for a subsonic inlet, as it allows the solver to calculate the resulting mass flow rate through the system. At the outlet, the static pressure was set to 1 bar, allowing the flow to exit the domain smoothly. Accordingly, a gradual development of the flow along the nozzle, where the fluid accelerates from a high-pressure, low-velocity state at the inlet to a lower-pressure, higher-velocity state at the outlet, driven by the pressure difference.

**Figure 3:**
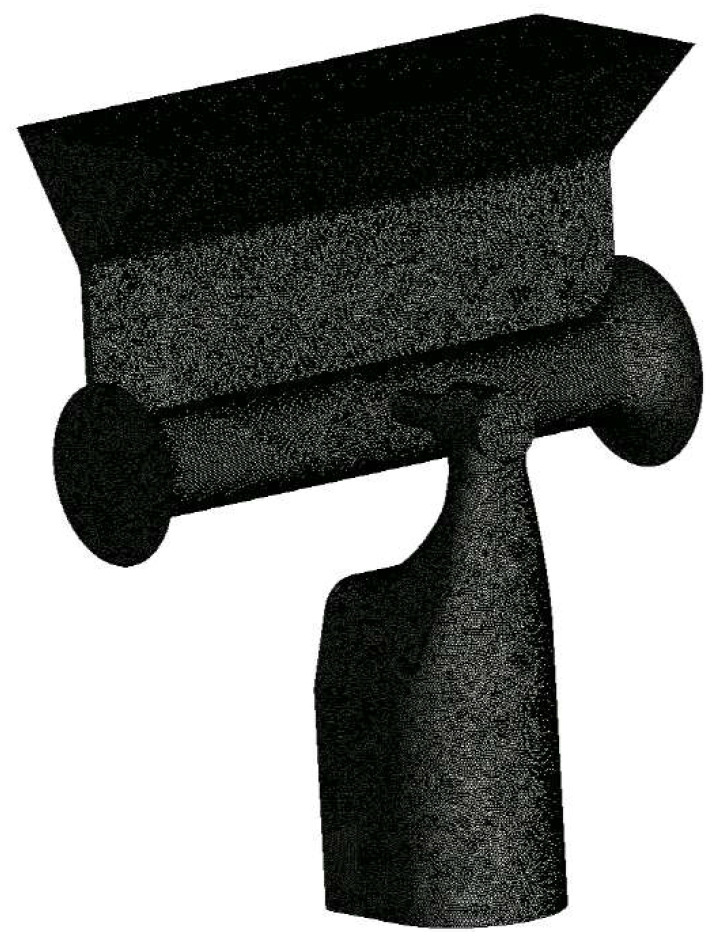
Typical mesh distribution.

**Figure 4:**
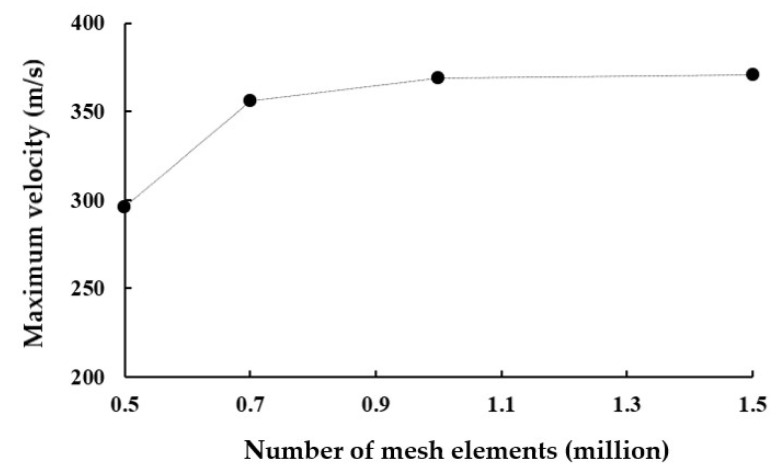
Mesh convergence test.

The computational domain was meshed using tetrahedral unstructured elements. The detailed mesh size was determined through a mesh convergence test, ensuring that the results are independent of the mesh size. For this mesh test, we used the baseline design with the circular channel with 0.6 mm air orifice. The total number of mesh elements in the computational domain varied from 0.5 million to 1.5 million. Figure 3 shows a typical mesh distribution obtained with 0.5 million elements. To enhance the accuracy of flow separation and vortex predictions, a higher mesh density was applied within the yarn channel and around the air orifice. The mesh dependence was assessed by comparing the maximum velocity in the yarn channel. Figure 4 shows the test results. There is little noticeable difference in maximum velocity between 1 million and 1.5 million. However, since many simulations need to be carried out for several geometric variations, the convergence index (GCI) [21,22] was also checked prior to extensive simulations.

The GCI evaluates quantitatively the uncertainty of simulation solutions. It is calculated using the following formula:

(1) $$GCI=F_{s}\frac{\left|\varepsilon \right|}{r^{p}-1},$$

where F_s_, p, and r indicate the safety factor of the method, the order of accuracy of the numerical method, and the grid refinement ratio, respectively. ε is determined as follows:

(2) $$\varepsilon =\frac{f_{coarse}-f_{fine}}{f_{fine}},$$

where f_coarse_ and f_fine_ are the numerical solutions obtained with a coarse and fine grid, respectively. Based on Roache’s recommendation [21], the safety factor F_s_ was set to 1.25. The GCI was calculated using three different number of elements, resulting in a GCI of approximately 4.3% for 1 million elements. Increasing the number of elements to 1.5 million decreased the GCI to 0.46%. Consequently, 1.5 million is a sufficient number of elements, considering its favorable GCI value and a balanced trade-off between numerical accuracy and computational cost. Specifically, the smallest and largest edge sizes were set to 0.0005 mm and 0.1 mm, respectively. For this computation, a personal computer equipped with 8-core 11th generation Intel(R) i7-11700 CPU and 32 GB RAM requires approximately 24 h.

## 4. Results and Discussion

### 4.1. Effects of the Cross-Sectional Shape of the Yarn Channel

The design of the yarn nozzle in air-jet spinning technology is crucial for producing high-quality yarns, characterized by well-separated and minimally damaged fibers [7]. In this study, two typical cross-sections of the yarn channel were simulated and compared: a square with rounded corners and a circular cross-section. Both the width of the square and the diameter of the circle are 2 mm, resulting in nearly identical cross-sectional area. For both cross-sections, the air orifice diameter was consistently set at 0.6 mm. Additionally, all simulations were performed under identical boundary conditions to ensure a fair comparison.

**Figure 5:**
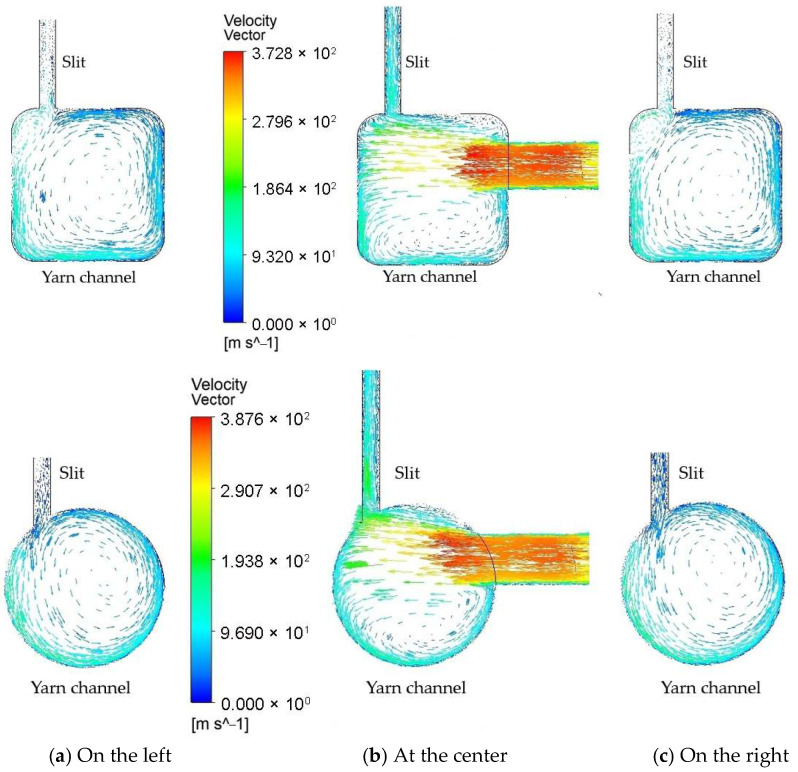
Comparison of the velocity vector on the cross-section of the yarn channel: (**a**) at 0.002 mm away to the left; (**b**) At the center of the yarn channel; (**c**) At 0.002 mm away to the right.

Figure 5 illustrates the velocity distribution across the cross-sections at three axial locations along the yarn channel: at the center, 0.002 mm to the left, and 0.002 mm to the right. At each location, well-developed vortex flows are observed, regardless of the cross-section shape. Notably, the primary vortex flow at the center of the yarn channel forms beneath the airflow from the air orifice, causing a significant portion of the supplied air to exit through the slit above. Consequently, the size and intensity of the vortex are greatly influenced by the airflow through the air orifice. The remaining airflow impacts the opposite wall of the yarn channel, developing into rotational flow along both sides of the yarn channel. This vortex airflow generally aids in migrating fibers in a helical form and reduces the hairiness of the spun yarn.

The vortices on the left and right sides are considerably larger, occupying almost the entire cross-section. This suggests that the vortex originates at the center and develops along both sides of the yarn channel. Although the overall flow patterns for the two different cross-section shapes appear similar, there are notable differences. In the square cross-section, a smaller counter-rotating vortex appears around the left upper corner, leading to unnecessary energy loss. Conversely, in the circular cross-section, a single strong vortex is formed.

**Figure 6:**
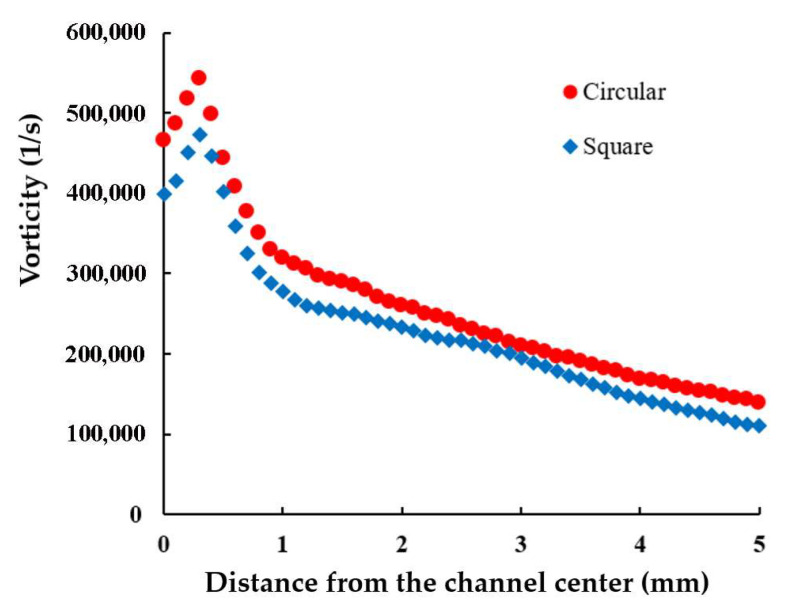
Comparison of the axial vorticity averaged on the cross-section along the yarn channel.

Figure 6 compares the axial vorticity averaged across the cross-section along the yarn channel’s direction. It shows that the vorticity is significantly influenced by the shape of the yarn channel, the vorticity with the circular shape being 7~26% higher than that with the square cross-section. This is an advantage of the circular cross-section over the square cross-section. Another thing to note is that the vorticity reaches its maximum near the edge of air orifice, regardless of the shape of the yarn cross-section.

### 4.2. Effects of the Diameter of the Air Orifice

The diameter of the air orifice is a crucial design parameter in the development of the yarn channel for air-jet spinning technology. This study explored the effects of varying the air orifice diameter on the air vortex flow within the yarn channel. Diameters ranging from 0.6 mm to 2.0 mm were investigated, considering the size of migration nozzles manufactured by CeraTrak [23]. Specifically, simulations were conducted for diameters of 0.6 mm, 1.0 mm, 1.3 mm, 1.5 mm, and 2.0 mm. All simulations were performed under identical boundary conditions, with the exception of the air orifice diameter size, to isolate its impact on the vortex flow dynamics.

**Figure 7:**
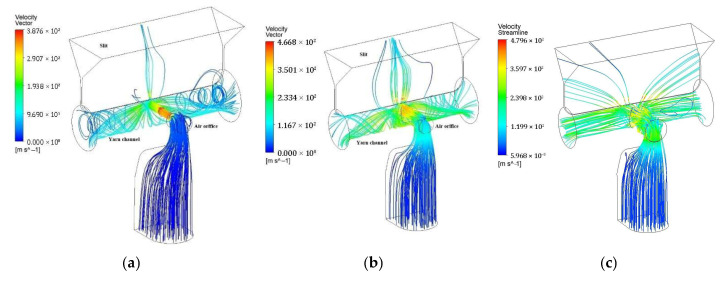
The streamline patterns starting from the inlet for three different diameters of the air orifice: (**a**) 0.6 mm; (**b**) 1.3 mm; (**c**) 2.0 mm.

Figure 7 illustrates the streamline patterns for air orifice diameters of 0.6 mm, 1.3 mm, and 2.0 mm. The maximum velocities reached are 387.6 m/s, 466.8 m/s, and 479.8 m/s for these respective diameters. A higher velocity magnitude indicates an increased airflow rate into the yarn channel, attributed to reduced flow blockage resulting from a larger air orifice diameter. This change in velocity magnitude significantly alters the vortex structure within the yarn channel.

One of the most notable effects of varying the air orifice diameter is on the swirling flow along the yarn channel. As shown in Figure 7, the swirling flow becomes noticeably weaker as the air orifice diameter increases. Another significant change in the flow pattern is the increase in airflow rate through the slit outlet with larger air orifice diameters. This means that more airflow exits through the slit rather than the ends of the yarn channel as the orifice diameter grows.

These findings align with the research conducted by Chau et al. [6], who noted that both insufficient and excessive inlet opening sizes result in fewer knots per unit length. Thus, the air orifice diameter has two opposing effects on the flow structure within the yarn channel: it reduces flow blockage, enhancing airflow into the channel, but also increases airflow leakage through the slit, weakening the swirling flow.

These observations suggest that there is an optimal air orifice size that maximizes the swirl motion within the yarn channel, balancing the benefits of increased airflow with the drawbacks of excessive leakage.

**Figure 8:**
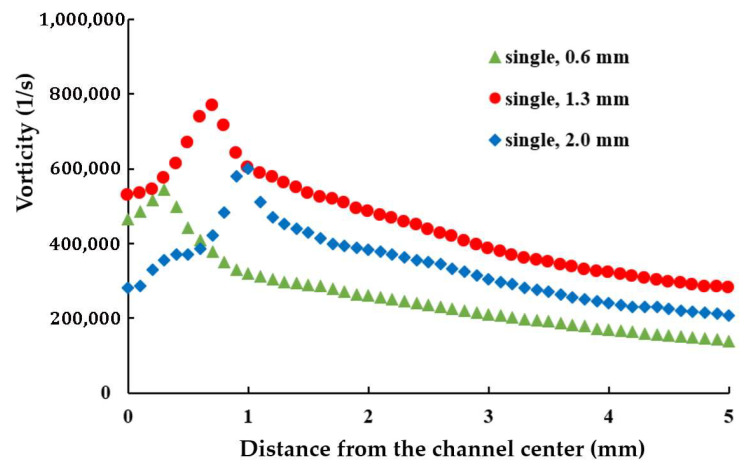
Effects of the diameter of air orifice on the axial vorticity averaged on the cross-section in the direction of the yarn channel.

To quantitatively evaluate the impact of the orifice diameter, the vorticity across the cross-section in the direction of the yarn channel was calculated. Figure 8 presents a comparison of the axial vorticity averaged across the cross-section for three different air orifice diameters. These results confirm that the averaged vorticity is significantly influenced by the orifice diameter. The maximum averaged vorticity is observed with an orifice diameter of 1.3 mm, suggesting that this diameter is optimal for achieving the best yarn quality given the current geometric and operational conditions of the yarn channel. This optimal diameter balances the advantages of enhanced airflow with minimized adverse effects, such as excessive leakage, thereby maximizing the swirl motion essential for high-quality yarn production. Figure 6 also confirms that the vorticity reaches its maximum near the edge of air orifice, regardless of the magnitude of the orifice diameter.

**Figure 9:**
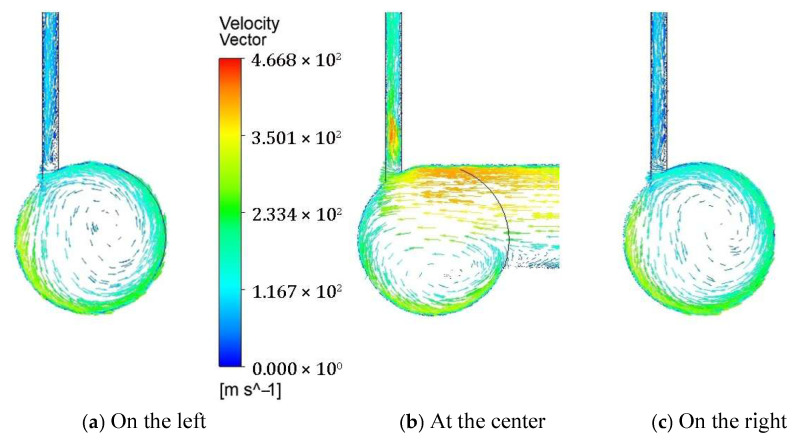
The velocity vector on the cross-section of the yarn channel at three locations along the channel for the air orifice of 1.3 mm diameter: (**a**) At 0.002 mm away to the left; (**b**) At the center of the yarn channel; (**c**) At 0.002 mm away to the right.

Figure 9 shows the velocity vectors on cross-sections at three axial locations along the yarn channel with an air orifice diameter of 1.3 mm: at the center, 0.002 mm apart to the left, and 0.002 mm apart to the right. Compared to the 0.6 mm diameter case depicted in Figure 5, the 1.3 mm diameter results in a significantly larger airflow rate exiting the yarn channel through the slit at all three cross-sections.

While a larger orifice diameter allows for a greater airflow rate into the yarn channel, it negatively impacts fiber twisting and increases the hairiness of the spun yarn. This comparison indicates that there might be other designs to improve the performance of the yarn channel by optimizing the vortex structure within the channel. While the 1.3 mm diameter with single air orifice offers some benefits, further refinement of the design could enhance yarn quality by balancing airflow and vortex dynamics more effectively.

### 4.3. Effects of the Number of the Air Orifice

To improve the performance of the yarn channel regarding the vortex structure within the channel, we investigated the effects of varying the number of air orifices. Generally, the number of air orifices is a critical parameter in designing yarn channels for air-jet spinning technology, although its impact on the vortex structure within the yarn channel is not yet fully understood. In this study, we conducted a comparative analysis of single and double air orifice designs. Aside from the number and diameter of the air orifices, all other geometric and boundary conditions remained consistent with those used in the previous simulations. This approach aimed to isolate the influence of the number of orifices on the vortex dynamics, providing insights into potential design optimizations for enhanced yarn quality.

**Figure 10:**
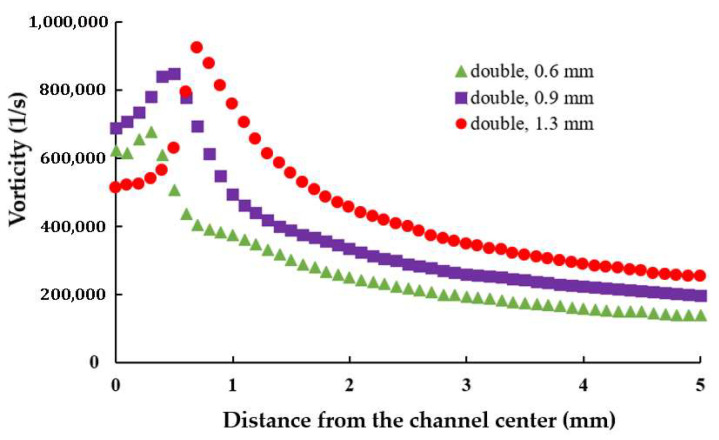
Effects of the diameter of air orifice on the axial vorticity averaged on the cross-section.

Figure 10 illustrates the variation in average axial vorticity along the yarn channel for three different air orifice diameters in a double orifice configuration. Compared to Figure 6, which represents the single orifice configuration, the vorticity distribution with double orifices is notably different. Specifically, with double orifices, a diameter of 0.9 mm achieves the highest vorticity in the air orifice area, while a diameter of 1.3 mm yields the highest vorticity outside the air orifice area. In the single orifice configuration, the maximum average vorticity was achieved with a 1.3 mm diameter.

**Figure 11:**
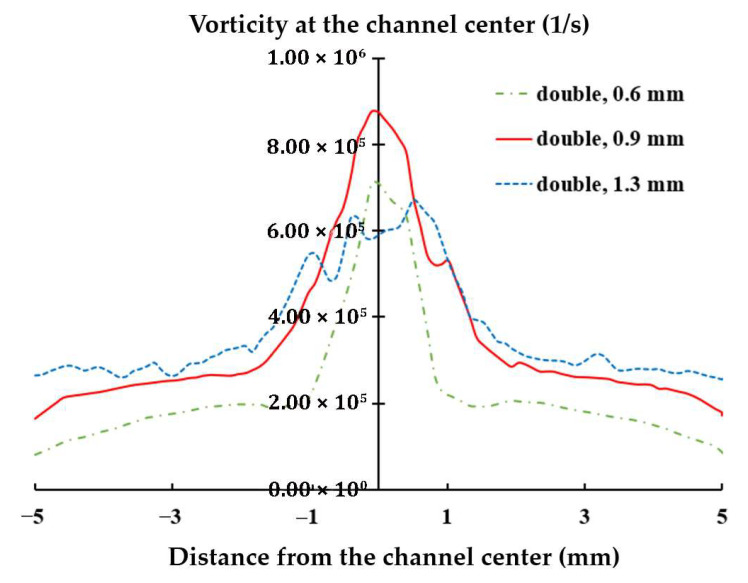
Effects of the diameter of air orifice on the axial vorticity at the centerline.

This finding suggests that optimizing the diameter of the air orifice should consider the number of orifices used. Figure 11 further compares the variation in axial vorticity along the centerline of the yarn channel, highlighting a strong dependency on the air orifice diameter, particularly in the area of air orifice. The diameter of 0.9 mm shows the best performance in terms of the centerline vorticity. Additionally, it is important to note that the centerline vorticity decreases rapidly along the yarn channel. In the case of 0.9 mm diameter, it drops by more than 60% within 2 mm from the center. Another thing to note is that there is a slight difference between the vorticities on the left and right. It helps the yarn movement inside the yarn channel.

The rapid decline in vorticity suggests that the vorticity in the air orifice area is crucial for enhancing yarn channel performance. By focusing on this region, the design can be fine-tuned to maximize the desirable vortex effects throughout the channel.

**Figure 12:**
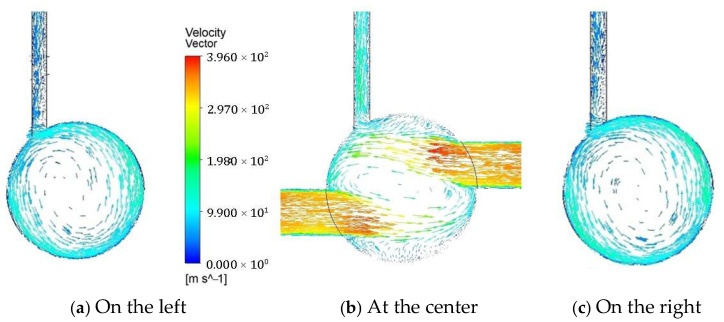
The velocity vector on the cross-section of the yarn channel at three locations along the channel with two air orifices: (**a**) at 0.002 mm away to the left; (**b**) at the center of the yarn channel; (**c**) at 0.002 mm away to the right.

Figure 12 illustrates the velocity vectors on cross-sections at three axial locations along the yarn channel with double air orifices of 1.3 mm diameter: at the center, 0.002 mm to the left, and 0.002 mm to the right. Compared to the single orifice configuration shown in Figure 7, a notable difference is that the center of vortex moved to the centerline of the yarn channel. In addition, it is energized by the two streams from the double orifices. Comparing Figure 6 and Figure 8, this flow pattern explains how the averaged vorticity for double air orifice is higher than that for single air orifice. The enhanced rotational airflow created by the double air orifice setup is beneficial for achieving improved yarn performance.

### 4.4. Experimental Validation

Air jet vortex spinning technology is widely recognized as a key flow feature that enhances the quality of spun yarn in the textile industry. Yarn produced using this technology undergoes significant structural changes, resulting in well-separated, straightened, and minimally damaged fibers [2,24,25,26,27]. In fluid dynamics, this vortex flow is quantified as vorticity. Thus, the effects of vorticity, as a critical parameter in the design assessment of migration nozzles, can be evaluated by examining the properties of the spun yarn. All design parameters should ultimately be evaluated from the perspective of the spun yarn. Consequently, the effects of increased axial vorticity are experimentally assessed based on yarn properties. To achieve this, yarn produced with two different nozzles was analyzed, focusing on two mechanical properties and three quality-related properties.

In this study, a migration nozzle developed by CeraTrak [23] was chosen as the basis design. This nozzle features a yarn channel with a diameter of 2.0 mm and a length of 10 mm. The test nozzles were manufactured from aluminum AES-11 by Sumitomo, Japan [28]. This material has a purity of 99.7% and a bulk density of 1.3 g/cm^3^. After manufacturing, hot isostatic pressing was applied as a surface treatment to eliminate any surface voids larger than 2 μm, at 1550 °C and 1000 bar.

**Figure 13:**
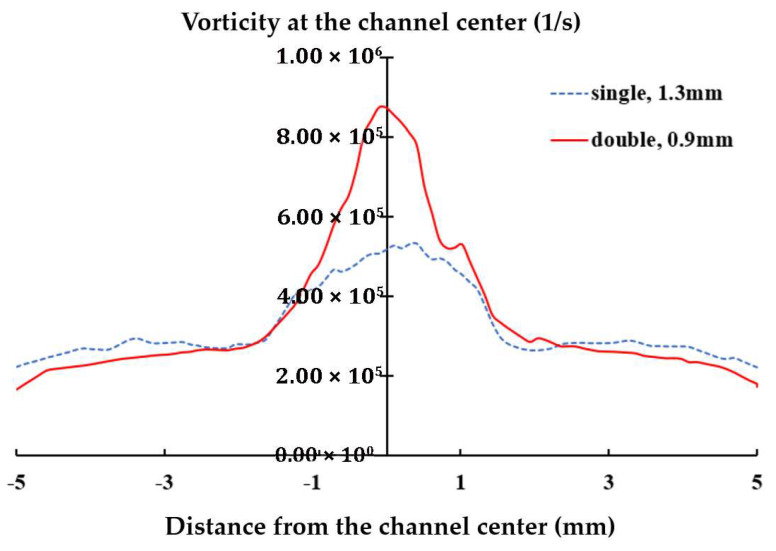
Comparison of the variation in the axial vorticity along the centerline.

The present CFD analysis confirms that a 1.3 mm diameter produces the highest vorticity along the centerline of the yarn channel, making it an optimal choice for single orifice configuration. However, for the double air orifice configuration, the diameter should be 0.9 mm based on the vorticity along the centerline in the air orifice area. Figure 13 compares the variations in axial vorticity along the centerline. The double air orifice with a 0.9 mm diameter exhibits a significantly higher vorticity at the centerline compared with the single orifice with a 1.3 mm diameter. The difference between the double 0.9 mm and single 1.3 mm configurations is most pronounced in the air orifice area. Higher vorticity is expected to result in improved yarn performance.

**Figure 14:**
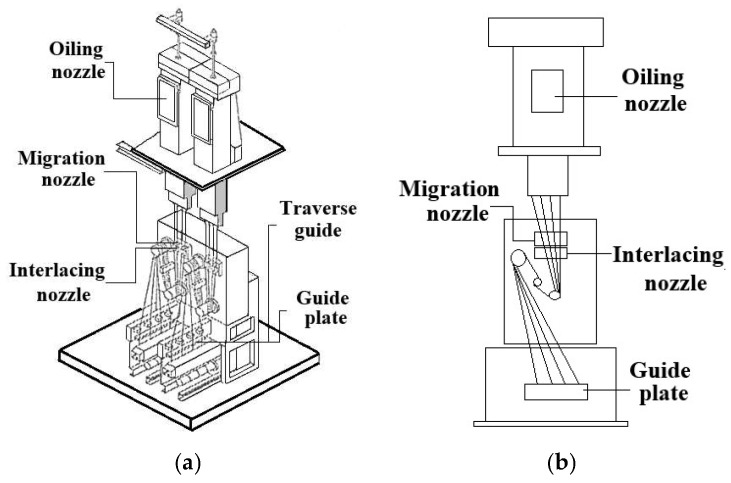
Schematic diagram of the experimental setup: (**a**) 3D view; (**b**) front view.

**Figure 15:**
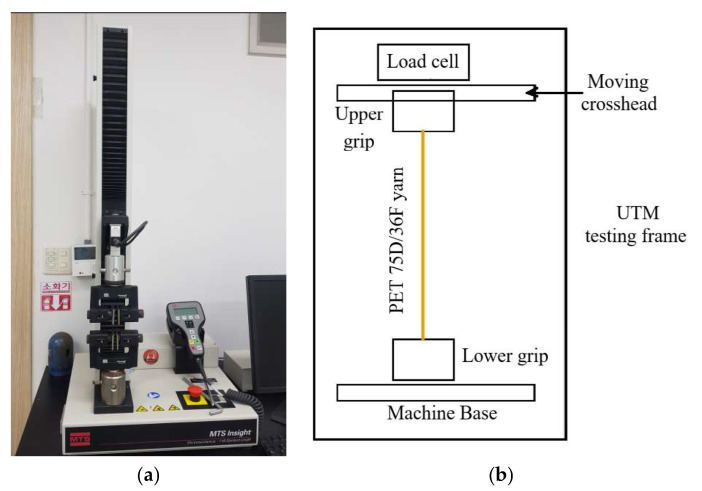
Mechanical property tester: (**a**) MTS Insight 1; (**b**) Schematic diagram.

For these two cases, polyester fiber PET 75D/36F was used, and the two migration nozzles were operated with the same interlacing nozzle [7]. Compressed air was supplied at a pressure of 3 bar, and the spun yarn was produced at a speed of 600 m/min. These experiment conditions represent a typical set of operation parameters for migration nozzles manufactured by CeraTrak [23]. Figure 14 shows a schematic diagram of the yarn experiment system, and Table 3 summarizes the experimental conditions. The manufactured synthetic fibers were evaluated based on two mechanical properties: tensile strength and elongation rate at break. For these measurements, a yarn string of 250 mm in length was stretched using a universal testing machine (UTM) equipped with suitable clamps. Figure 15 shows the UTM used in this study. This UTM is an electromechanical device that applies tensile force until the fabric breaks, measuring the corresponding strength and elongation rate at break. Tensile stress measurement was performed in accordance with ASTM D2256 (Standard Test for Tensile Properties of Yarns by the Single-Strand Method) [29]. For each test, a yarn specimen of 250 mm was mounted on pneumatic clamps and was stretched at a constant rate of extension until rupture. Specimens were tested in a standard textile atmospheric condition of 20 °C and 65% relative humidity (RH). The crosshead speed was set to achieve an average time-to-break 20 s as specified in D2256, equivalently 300 mm/min at 250 mm gauge. Pre-tension of 0.5 cN/tex was applied to remove slack before the run. Break was detected when the real-time force dropped by 40% within 50 ms. The UTM (MTS Insight 1 [30]) records both the maximum tensile force and the corresponding elongation rate at break. Generally, higher values of elongation rate and tensile strength indicate improved flexibility and/or filament twisting of the yarn. In addition to these mechanical properties, yarn quality is also assessed in terms of knot number, hairiness and migration index. The number of knots was manually counted for each yarn sample. There is not one single international standard protocol used exclusively for counting knots in spun yarn, it is typically addressed as part of a larger quality control process.

**Figure 16:**
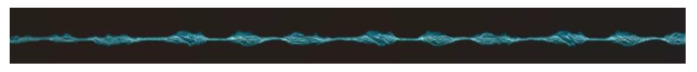
An example of the spun yarn with several knots.

When counting knots, the number of knots in a given length of yarn, such as a meter or an inch, is a traditional measure [31]. In this study, the number of knots in a 1 m length of the spun yarn was counted repeatedly three times per sample by two different engineers. The average value was then recorded. Figure 16 illustrates an example of the spun yarn with several knots.

Hairiness was measured using a Uster hairiness tester [32,33] under the standard textile atmospheric condition of 20 °C and 65% relative humidity (RH). The tester uses a series of optical sensors (OH module) to scan the yarn and calculates the hairiness value (H value) by analyzing the yarn, providing a basis for comparison with global benchmarks and yarn trading standards. The measurement was repeated twenty times, with data collected three times for each measurement after a short break, ensuring randomization of data. Detailed information on these testers is provided in Table 4.

The fiber migration index (MI) was determined as the average number of exchanges between sheath and core fibers per 100 mm of yarn using the dye tracer method. In this method, the sheath and core filaments are dyed different colors, and their movement is counted using a microscope by different engineers, recording the mean value. Specifically, the sheath fibers were dyed black while the core fibers were white. Using a microscope, the points where the color changes were observed, and the number of exchanges between sheath and core fibers was counted.

Due to experimental uncertainties from factors like manufacturing tolerances and measurement errors, we conducted a statistical analysis of all data. To determine a reasonable number of specimens, we carried out a power analysis using G*Power 3.1 [34]. With α (significance level) = 0.05, power = 0.8, and d (effect size) = 0.85, at least eighteen specimens were needed. To do this, a sufficient number of specimens were collected from the spun yarn produced with the two migration nozzle settings. From this collection, we randomly selected twenty yarn specimens (n = 20) and tested. One engineer produced more than twenty specimens without specifying their intended use. Each specimen was marked with a different number of dots. Another engineer conducted testing without being informed of the markings. The results, summarized with mean and standard deviation in Table 5, were analyzed using a one-way ANOVA with Tukey’s post hoc test. The *p* values indicate a significant improvement in the four properties including mechanical properties. As the centerline vorticity increased by 62% from 5.03 × 10^5^ (1/s) to 8.16 × 10^5^ (1/s), the tensile strength rose from 3.2 N to 3.7 N, marking a 15.6% improvement. Similarly, the elongation rate at break improved from 19% to 22%, a 15.8% increase. Both improvements are statistically significant (*p*-value < 0.05). The number of knots per meter increased from 23.21 to 24.01, a 3.4% increase that is also statistically significant (*p*-value < 0.05). The migration index also increased from 0.57 to 0.63, a 10.5% increase that is also statistically significant (*p*-value < 0.05). Therefore, the two mechanical properties exhibit a positive correlation with the centerline axial vorticity, with a slope of 1/4 while the migration index shows a slope of 1/6. A slight improvement was obtained in the number of knots per meter. We also analyzed the effect size by calculating Cohen’s d value using $\left|M_{1}-M_{2}\right|/s$. Here, M_1_ and M_2_ are the means of the single air orifice data and double air orifice data, respectively. The pooled standard deviation, s, was calculated as $s=\sqrt{\left(n_{1}-1)sd_{1}^{2}+(n_{2}-1)sd_{2}^{2}/(n_{1}+n_{2}-2\right)}$. n_1_ and n_2_ are the numbers of specimens for the single air orifice and double air orifice, respectively. sd_1_ and sd_2_ are the standard deviations of two specimens 1 and 2. The values for tensile strength, elongation at break, knots per meter and migration index are 1.41, 2.46, 1.15, and 1.29, respectively. These indicate that the four properties are not only statistically significant but also practically meaningful. However, the improvements in these properties are 15.6%, 15.8%, and 10.5%, respectively. This suggests that both the single and double air orifice designs are effective, as they are already optimized for the diameter of the air orifice. The design change based on the centerline axial vorticity led to further enhancement of the spun yarn properties. In contrast, the hairiness showed only slight improvements that are not statistically significant (*p*-value > 0.05). The hairiness index also shows the smallest effect size.

These distinctive improvements in both mechanical properties, number of knots per meter and yarn migration index, coupled with the significant difference in the centerline vorticity between the double 0.9 mm and single 1.3 mm configurations, suggest that the axial vorticity along the centerline is a crucial and practical parameter for the design assessment of migration nozzles.

## 5. Conclusions

Migration nozzles are extensively utilized in the post-spinning stage of synthetic fiber production and typically designed and manufactured according to customer specifications. This paper presents a practical design analysis approach aimed at enhancing the performance of migration nozzles through the use of CFD simulations. The present design approach evaluates the flow characteristics within the yarn channel by analyzing the vortex flow and calculating the vorticity along the centerline of the yarn channel.

A commercial migration nozzle manufactured by CeraTrak [23] was chosen as the baseline design. The performance of this nozzle was simulated with a focus on the vortex flow characteristics within the yarn channel. To enhance its performance, three major design parameters were examined by varying their values: the cross-sectional shape of the yarn channel, the diameter of the air orifice, and the number of air orifices.

The cross-sectional shape of the yarn channel significantly influences the formation of vortex flow within the yarn channel. In this paper, both square and circular cross-sections were analyzed. For the square cross-section, a secondary vortex was observed above the main vortex, leading to unnecessary energy loss. In contrast, the circular cross-section exhibited higher vorticity along the centerline, making it preferable over the square cross-section.

The diameter of the orifice significantly affects the swirling motion of the vortex flow within the yarn channel. To investigate this effect, five different orifice diameters from 0.6 mm, 1.0 mm, 1.3 mm, 1.5 mm, to 2.0 mm, were simulated. The results indicate a strong dependence of centerline vorticity on the diameter of the air orifice, with the 1.3 mm diameter yielding the highest vorticity along the centerline in the case of single air orifice.

CFD simulations were conducted further to evaluate the effects of the number of air orifice on the axial vorticity along the centerline. Two cases, a single orifice and a double orifice, were simulated. For the double air configuration, three different diameters were simulated: 0.6 mm, 0.9 mm and 1.3 mm. The simulation showed that the 0.9 mm diameter produced the highest centerline vorticity for the double air orifice configuration. In total, five single air orifice and three double air orifices were compared with each other. The vorticity was evaluated in two different ways: one is the vorticity averaged on the cross-section in the direction of the yarn channel and the other is the vorticity at the centerline.

The centerline vorticity in the area of the air orifice, when compared with the corresponding experimental results, correlated with the two nozzle geometries we tested. It serves as a key and reliable parameter for evaluating the design of migration nozzles. For both simulation and experimentation, we examined a single set of typical design conditions for migration nozzles, even though they are expected to operate with some variations in the field. We simplified this study by neglecting factors such as the surface roughness of migration nozzles, oil mist, and variations in operational conditions like speed and air pressure.

## Figures and Tables

**Table 1:** Characteristics of the baseline migration nozzle system.

	Yarn Channel	Air Orifice	Slit Outlet
Shape	Square	Circle	Rectangle
Length (mm)	12	1.7	12
Width **×** height (mm)	2 × 2	Diameter = 0.6 mm	4.0 × 5.0

**Table 2:** Boundary conditions.

Boundary	Location	Value
Air inlet	Air in	3 bar
Air outlet	Both ends of the yarn channel	1 bar
Opening	Yarn loading slit	1 bar
Wall	Nozzle body	No-slip

**Table 3:** Experimental conditions.

Air Orifice, Diameter (mm)	Yarn	Air Pressure (bar)	Testing Speed (mm/min)	Gauge Length (mm)
Single, 1.3	PET 75D/36F	3.0	500	250
Double, 0.9	PET 75D/36F	3.0	500	250

**Table 4:** Yarn property testers.

	Mechanical Property Tester	Hairiness Tester
Manufacturer	MTS	USTER
Model	Insight 1	5-s800
Load/Test time	Load 1 (kN)	Test time 10 min
Testing speed	0.001–1500 (mm/min)	Up to 800 m/min

**Table 5:** The experiment results (mean ± standard deviation)/95% confidence range.

Property	Single, 1.3 mm /95% Confidence Range	Double, 0.9 mm /95% Confidence Range	*p*-Value /Cohen’s d
Tensile strength (N)	(3.22 ± 0.28) /3.12~3.33/	(3.57 ± 0.21) /3.49~3.65	0.005 /1.41
Elongation at break (%)	(19.21 ± 1.46) /18.67~19.76	(22.21 ± 0.92) /21.87~22.55	0.00003 /2.46
Knots per meter	(23.21 ± 0.65) /22.97~23.45	(24.01 ± 0.74) /23.73~24.29	0.019 /1.15
Hairiness index	(5.07 ± 0.27) /4.97~5.17	(4.98 ± 0.32) /4.86~5.61	0.51 /0.30
Migration index (MI)	(0.53 ± 0.07) /0.50~0.56	(0.62 ± 0.07) /0.59~0.65	0.010 /1.29

## Data Availability

The original contributions presented in this study are included in the article. Further inquiries can be directed to the corresponding author.

## Abbreviations

The following abbreviations are used in this manuscript:

CFD	Computational Fluid Dynamics
UTM	Universal Testing Machine
